# The Immune Response and Its Therapeutic Modulation in Bronchiectasis

**DOI:** 10.1155/2012/280528

**Published:** 2012-10-10

**Authors:** Massoud Daheshia, James D. Prahl, Jacob J. Carmichael, John S. Parrish, Gilbert Seda

**Affiliations:** Department of Pulmonary Medicine, Naval Medical Center San Diego, San Diego, CA 92134, USA

## Abstract

Bronchiectasis (BC) is a chronic pulmonary disease with tremendous morbidity and significant mortality. As pathogen infection has been advocated as a triggering insult in the development of BC, a central role for the immune response in this process seems obvious. Inflammatory cells are present in both the airways as well as the lung parenchyma, and multiple mediators of immune cells including proteases and cytokines or their humoral products are increased locally or in the periphery. Interestingly, a defect in the immune system or suppression of immune response during conditions such as immunodeficiency may well predispose one to the devastating effects of BC. Thus, the outcome of an active immune response as detrimental or protective in the pathogenesis of BC may be dependent on the state of the patient's immunity, the severity of infection, and the magnitude of immune response. Here we reassess the function of the innate and acquired immunity in BC, the major sites of immune response, and the nature of the bioactive mediators. Furthermore, the potential link(s) between an ongoing immune response and structural alterations accompanying the disease and the success of therapies that can modulate the nature and extent of immune response in BC are elaborated upon.

## 1. Introduction

Bronchiectasis (BC) is clinically characterized as irreversible dilation of the bronchi and bronchioles, leading to persistent cough, purulent sputum, and decreased airway flow, which can be accompanied by recurrent exacerbations. Disease detection has greatly advanced with the advent of high-resolution computed tomography (HRCT). However, management of the disease and treatment options are not yet fully optimal, and at present there is no disease-modifying treatment available for BC [1].

One major reason for this suboptimal treatment of the disease is the lack of conclusive evidence regarding the underlying cellular and molecular characteristics of BC, especially the role of the immune response. On one hand, multiple hematopoietic cells and their bioactive cellular and humoral products have been detected during expression and persistence of the disease, along with a positive association between BC and a series of chronic inflammatory diseases such as rheumatoid arthritis. On the other hand, a set of immunodeficiency disorders or subtle defects in the generation of a normal immune response have been shown to predispose an individual to development of BC [1]. Although no antigen specificity has been reported in BC, increased lymphocytes have been shown in submucosal and epithelial layers of the airway, as well as increases in specific classes of blood immunoglobulin, which point to the notion that both innate and acquired immunities are implicated in BC. In this report, the influence of the immune response and its secreted products are evaluated in the context of structural modification and clinical manifestations of BC. 

## 2. Inflammatory Cells in Bronchiectasis

Neutrophils (PMN), macrophages, natural killer (NK), mast cells, and lymphocytes have been identified during different stages of BC [2–6]. A greater number of T cells, B cells, NK cells, and macrophages exhibiting activation markers were shown in the bronchial lamina propria of BC patients (*n* = 22) compared to controls (*n* = 11) [6]. Tissue biopsies from BC patients confirmed an increased T cell infiltration in the epithelium, lamina propria, and submucosa, with CD8^+^ cells as the main T cell subset [7]. However, neutrophils are suggested as the most abundant cells infiltrating into BC pulmonary tissues, and therefore some have called BC a disease of neutrophilic inflammation of the lung [8]. Increased numbers of neutrophils in bronchioalveolar lavage (BAL) fluid were reported in noncolonized stable BC patients (*n* = 23) versus controls, and the BAL neutrophilia was significantly exaggerated in BC patients with microorganisms with potential pathogenicity (MPP) (*n* = 22) [9]. Elevated numbers of neutrophils were shown in sputum [10] and BAL [9, 11], as well as in lung parenchyma [2] of BC patients. Zheng et al. reported the presence of PMN in the airway lamina propria of BC patients, where neutrophil density negatively correlated with FVC% [2]. In the same study, increased macrophages in the BC sputum were suggested as the contributing factor to neutrophil influx into airway wall through production of TNF*α* [2]. Pulmonary TNF*α* along with IL-8 were also suggested as the main neutrophil chemotactic mediators [12]. Experiments with radiolabeled granulocytes demonstrated that pulmonary neutrophils migrated from the bloodstream into the bronchiectatic areas of lungs of BC patients, and about half of the migrated neutrophils would be lost via sputum [13].

These data suggest that a broad active cellular immune response including almost all types of hematopoietic cells is present during the expression of BC.

## 3. Inflammatory Mediators in Bronchiectasis

Diverse classes of inflammatory molecules, including proteases, free radicals, or cytokines, with different sets of biochemical activities have been implicated in pathogenesis of BC (Table 1). The presence of these mediators has led several investigators to propose the hypothesis of a specific pulmonary milieu that could favor the development of BC. Chan et al. suggested a specific pro-protease environment where, due to a formed complex with syndecan-1, activities of proteases, such as neutrophil elastase, remain unopposed [14]. soini et al. showed that tumor-associated trypsin inhibitor (TATI), a specific endogenous trypsin inhibitor, was increased in BC BAL fluid causing an imbalance in the proteinase/antiproteinase, which culminated in a significant increase in BAL MMP-8 and -9 of BC patients versus healthy controls [15].

Free radicals are another class of key factors associated with BC. Increased pulmonary extravasation of neutrophils and macrophages, along with an activated epithelium may well be the cellular sources of augmented free radicals [16, 17]. There are reports suggesting that increased free radicals may be more than just surrogate biomarkers of inflammation. Studying 53 patients with stable BC and 20 patients during BC exacerbation, Shoemark et al. reported an increased exhaled nitric oxide (NO) that reflected the severity of the disease [16]. Loukides et al. showed a significant increase in exhaled H_2_O_2_ levels of patients with stable BC (*n* = 30), which positively correlated with percentage of neutrophils in induced sputum, the extent of the disease as defined by HRCT, and impairment of lung function as measured by FEV1% predicted [17]. As the severity scores of HRCT have also positively correlated with TNF*α* and IL-8 levels present in the sputum in children with BC (*n* = 27) [10], it is well conceivable that the clinical outcome of BC are due to the cooperative actions of multiple factors. 

## 4. Systemic Immune Activation in Bronchiectasis

Although not studied as much as pulmonary inflammation during BC, an active systemic immune response (hyperimmune response) has also been reported in BC patients [18–20]. Studying 120 BC patients Wilson et al. showed an elevation of blood neutrophils and total cell counts as well as increased erythrocyte sedimentation rate, which all correlated with the extent of disease and poor lung function [18]. Ip et al. demonstrated increased total peripheral white blood cells (WBCs) including neutrophils and lymphocytes in BC patients (*n* = 85) versus controls, and a negative correlation between FEV1 % predicted and WBC count [19]. Serum concentration of total IgG, its subclasses, IgM, and IgA tended to be higher in BC patients (*n* = 23) versus control, with the difference in IgA being significantly higher (*P* < 0.01) [20].

Systemically, increased inflammatory mediators were also shown in BC [18, 21–23]. An ongoing acute phase response including both C-reactive protein (CRP) and serum amyloid A (SAA) was elevated in blood of BC patients [18, 23] where an association between peripheral level of CRP and a greater pulmonary functional deterioration was shown [21]. Among the end-stage respiratory disease patients, BC patients had the highest prevalence of elevated blood CRP, which appeared as major determinants of hospitalization and risks of death [24]. Simultaneously, elevated levels of TGF*β* in the serum of patients with stable BC have been postulated as the orchestrator of subepithelial fibrosis associated with chronic airway inflammation in BC [22].

In the study by Zheng et al. the authors demonstrated an upregulation of serum adhesion molecules such as ICAM-1, VCAM-1 and E-selectin in BC patients, which is probably a reflection of increased systemic inflammatory cells [25]. ICAM-1 levels correlated positively to 24-hour sputum volume, and both E-selectin and ICAM-1 showed association with the number of pulmonary lobes affected by BC, and correlated negatively with FEV_1_ and FVC% predicted [25]. 

These data indicate that BC is a progressive disease with peripheral ramifications such as systemic inflammatory response; which has also been shown in asthma or COPD. At present it is not clear whether the systemic inflammation is an active inducer of BC or rather an indication of its clinical severity and state. However, it has been shown that BC patients with high plasma concentrations of TNF*α* had more severe disease, were more likely to have respiratory failure, and a higher rate of bacterial colonization [26]. Furthermore, the heterogeneity of BC may be highlighted by the study of Arsava and Coplu, which showed a significant increased in systemic inflammatory parameters only in the BC patients with persistent colonization, but not in BC patients with stable condition [27].

## 5. Inflammation and Pulmonary Structural Alterations

Structurally, BC has been characterized as severe deformation of bronchial wall components with inflamed dilated and thick-walled bronchi, with the presence of lymphoid follicles in the bronchi [1, 28]. As reported by several investigators, the influx of inflammatory cells into the lung parenchyma leads to altered airway structure, which potentially facilitates airway bacterial colonization, reduction in ciliary functions and subsequent reinfections [2, 5, 29]. Shum et al. showed that neutrophils activated by bronchial secretion from patients with BC, containing TNF*α* as a major component, were able to degrade the proteoglycans, an important component of the lung extracellular matrix (ECM) [30]. Studying BAL fluid from 33 BC patients, Sepper et al. demonstrated that the increased MMP-8 and -13 contributed to degradation of type 1 collagen, another abundant structural components of the lung ECM, causing the loss of functional integrity of the lung [31]. Although there was no obvious difference in the number of mast cells in the BC lungs versus controls, the presence of degranulated mast cells was more evident in BC lung tissue, and a significant correlation was observed between levels of tryptase, a component of mast cell degranulation, and BC disease severity [4]. In this context, immune cells, both residents as well as extravasated cells, may be an explanation for the chronic and progressive nature of BC. 

Alternatively, the inflamed and thick-walled bronchi can also be explained by infiltration of inflammatory cells into the mucosal BC tissues [3]. Gaga et al. obtained mucosal biopsy specimens from 12 BC patients and reported, in addition to neutrophilia, an intense mononuclear cell infiltrate including monocytes and lymphocytes [3]. Studying tissue from 9 patients with BC, Lapa e Sliva et al. showed that inflammatory cells in the bronchial wall of patients with BC were almost exclusively mononuclear cells, with T lymphocytes as the major cell type [7]. Neutrophils were further associated with additional BC symptomatic changes such as chronic purulent sputum production, a usual feature of BC [32]. Fahy et al. reported that BC sputum contained marked secretagogue activity, most of which was due to neutrophil proteases [33]. 

In the study by Sykes et al. it was shown that serine proteases present in purulent sputum are one of the key factors impairing human ciliary beating and function [34], which has previously been reported by Smallman et al. using sputum from BC patients [35]. Stockley et al. demonstrated that sputum color as well as sputum volume positively correlated with inflammatory cells present in lung of BC patients [36, 37]. The same investigators reported that the increase in air trapping (ratio of residual volume/total lung capacity) was closely related to purulence and elastase content of sputum [37]. It has routinely been shown that the increased pulmonary products of immune cells, mainly neutrophils' and macrophages' elastases and metalloproteases, result in goblet cell hyperplasia and mucus hypersecretion [38].

These results can suggest that inflammatory cells and/or their bioactive secretory mediators may be at least partly responsible for the structural alterations commonly linked to the pathogenesis of BC.

## 6. Immunoinflammatory and Autoimmune Disorders Associated with Bronchiectasis

A preexisting inflammatory disorder, such as rheumatoid arthritis (RA), is an etiologic factor for the development of BC [39]. Using sensitive HRCT, BC has been revealed in 20–35% of patients with RA [40], and 58% of patients with early RA exhibited BC on CT [41]. Although the cause-effect relationship is not clear, multiple inflammatory disorders—many with extrapulmonary manifestations—have been associated with BC, in such extent that BC has been considered to include a possible autoimmune component [42]. Generation of autoantibodies, autoreactive T cells, new antigenic epitopes, common detrimental mediators, and genetic polymorphisms in immunological factors may be considered as potential mechanisms of BC development. Demoruelle et al. showed that 76% of subjects possessing RA-related autoantibodies exhibited airway abnormalities including BC [43]. Popler et al. reported a case of a patient with autoimmune polyendocrine syndrome type 1 exhibiting autoantibodies directed at the potassium channel regulatory protein who exhibited significant lymphocytic infiltrates of the airways on lung biopsy and had findings of BC on thoracic imaging [44]. Circulating autoantibodies to bactericidal/permeability-increasing protein (BPI), one of the major PMN antimicrobial proteins, was also reported for BC patients [45].

A subset of patients undergoing colectomy for Crohn's disease has been shown to develop BC post surgery [46]. Eaton et al. suggested generation of a new epitope subsequent to the surgery, which consequently becomes the target of immune system, as an explanation for the development of BC [46].

BC is present in more than 50% of COPD patients, and shared anatomical, immunopathological, and clinical features in BC and COPD have been suggested as the underlying causes of this common overlap [47]. Severe airflow obstruction, a positive culture of microorganisms with potential pathogenicity (PPM) from sputum, increased immune cell infiltration and proteolytic factors in the lung and during acute exacerbations share common overlapping characteristics of both diseases [47].

 It has been reported that functional polymorphisms in IFN*γ*, neutrophil chemokine CXCR1, or a combination of both are associated with 5.6-, 8.3- and 56-fold increased susceptibility to BC-associated UC, respectively [48]. The genetic alterations in mediators of immunity and BC may be emphasized by the data that HLA-Cw*03 alleles, HLA-C homozygocity, as well as the HLA-DR1, DQ5 haplotype are associated with increased susceptibility to BC [49, 50].

Therefore, multiple inflammatory diseases, through a wide variety of mechanisms causing exaggerated immunpathological responses, can be associated with BC development (Table 2).

## 7. Immunodeficiency and Altered Immune Response as Risk Factors for BC

Patients with primary or secondary immunodeficiency are more susceptible to the development of BC as well as individuals with defects in generation of an adequate immune response [51] (Table 3). In a retrospective study of 164 HIV-I infected children it was shown that more than 15% developed BC, with a strong association between BC and deterioration of immune status and advanced stage of HIV infection, that is, CD4^+^ T cells <100 cells per cubic millimeter [52]. It has been shown that 80% of patients with primary immunodeficiency, due to a defect in transporter associated with antigen presentation (TAP), have recurrent bacterial infections of respiratory tract and BC [53]. Furthermore, lack of expression of HLA-A, HLA-B, and HLA-C class I antigens on the lymphocytes (the bare lymphocytes syndrome) may predispose a subject to BC [54], and HLA class I antigen deficiency has been shown associated with familial BC [55].

A major role for the humoral immune response is concluded from studies demonstrating that more than 37% of BC patients had defects in antibody-mediated immunity [56] and more than 12% of BC patients had decreased levels of at least one of the IgG [21]. BC has been observed in 42–73% of common variable immunodeficiency (CVID) patients, affected by a marked reduction of IgG, IgA, and/or IgM [57], and in whom immunoglobulin replacement is an important tool to avoid the progression of BC [58]. A defect in memory B cells, responsible for a rapid and efficacious onset of specific antibody production, has been shown to cause the development of BC in 50% of patients [59]. Interestingly, an intravenous immunoglobulin delivery to CVID patients with BC was associated with a significant decrease in exhaled NO, sputum inflammatory cell counts, and a major increase in respiratory mucus transportability by cough [58]. 

A correlation between incidence of BC and severity of immunodeficiency is stressed by Rivoisy et al. who reported that BC in CVID patients increased from 29% to 58% if CVID patients had also a decreased T cell activity due to a parental consanguinity [60]. The influence of compromised immunity on the outcome of therapy of BC patients was highlighted by Hiramatsu et al. as an immunocompromised state was shown to be a statistically significant and independent factor that had an adverse impact on the surgical treatment of BC [61]. Furthermore, the immunosuppressor drugs, mainly used following organ transplantations, have shown to induce BC by long-lasting negative effects on the quality of the immune response [62, 63].

Immune deregulation has also been implicated in BC. More than 25% of individuals seropositive for human T cell lymphotropic virus types 1 (HTLV-1) in an indigenous Australian population were diagnosed with BC [64], and HTLV-1-seropositive patients were significantly more likely to die from a BC-related complication [65]. The pathologic correlate of these observations is a lymphocytic infiltration of bronchiole walls and mucosal glands, which may lead to insufficiency of respiratory tract mucus secretion [66], and predispose patients to recurrent or persistent respiratory infections, thereby increasing the likelihood of progression to BC [64].

These data may point to the protective side of immunity in BC where an insufficient immune response will allow more frequent and vigorous infections causing tissue damage and lead to the persistence of pathogen or colonization. This notion may be supported by the positive results of vaccinations as major contributors of decreased BC in developed countries [67].

## 8. Discussion

The involvement of immune response in BC is at least twofold. In one hand a lack of adequate immune response at the time of infection may make one more susceptible to respiratory infections and subsequent BC. This may also explain why the most common cause of BC in the literature is postinfectious complications in early childhood, when the immune system is possibly not yet completely established [1], and why the incidence of BC increases in adults over 75 years, in whom the strength of immune response has substantially deteriorated [68]. On the other hand, an exaggerated immune response, for example, due to a high microbial load, may damage lung tissues and provide permanent niches for the pathogens, from where the potential microbial spread may pursue. Therefore, both inadequate as well as exaggerated immune response may contribute to the chronic phase of the disease, a pathologic state where proteolytic mediators, proinflammatory molecules, and high mucus secretion cannot only injure the pulmonary structures, but also delay the pathogen clearance. This dual role of immune response in BC, partially because of the timing and strength of infection, along with patients' health status, may be an explanation as to why the effectiveness of anti-inflammatory agents, such as steroids, toward the management of BC has been inconclusive [69]. 

As several inflammatory diseases are associated with BC, to have these comorbidities under control may be an effective part of the BC management. Existing examples include steroid treatment of BC patients with allergic bronchopulmonary aspergillosis (ABPA) and immunoglobulin therapy of BC patients with CVID. However, RA patients with concomitant BC, treated with biologic disease modifying drugs, such as anti TNF*α*, are at risk of lower respiratory tract infection [70], stressing the view that consequences of treatment of primary disease on the outcome of BC has to be carefully evaluated.

Both severity of infection and state of an individual's immunity are the key factors involved in BC. In addition, it is well conceivable that immunopathological or immune-autoreactive conditions create favorable conditions and encouraging milieus for the development of BC, or at least set the tone for its induction following pulmonary infections. Existing data suggest that BC is not a homogenous disease, with multiple initiation pathways and progression branches. Limited clinical trials, lack of a validated biomarker, and absence of a well-established animal model of the disease have been a few setbacks in BC management. 

 Hence, based on our current knowledge, therapeutic management should aim at a comprehensive approach to generate a minimum effective immunity to clear the pathogen and blocking lung colonization without creating immunopathological conditions. Therefore, personalized disease management along a combinational therapy, including immunomodulatory modalities, may be an optimal option. In one hand, a pathogen-specific immune response should be boosted prior to or at onset of infection, as in the case of active or passive immunization, and on the other hand the unwanted and unsatisfactory immune responses should be hampered during chronic phase of immune response to limit pulmonary damage. 

## Figures and Tables

**Table 1:** Proinflammatory and immune mediators upregulated during bronchiectasis.

Class of mediators	Type of mediators
Cytokines	TNF*α*, IL-1*α*, IL-1*β*, IL-6, IL-8, TGF*β*
Proteases	Neutrophil elastase, MMP2, MMP8, MMP9, MMP-13, Cathepsin B, Cathepsin G, mast cell tryptase
Leukotrienes (LT)	LTB4
Adhesion molecules	ICAM-1, E-selectin, VCAM-1
Granule proteins	Myeloperoxidase
Acute-phase proteins	C-reactive protein (CRP), serum amyloid A (SAA)
Free radicals (exhaled)	NO, H_2_O_2_

**Table 2:** Inflammatory diseases associated with the pathogenesis of bronchiectasis.

Rheumatoid arthritis
Ankylosing spondylitis
Relapsing polychondritis
Sarcoidosis
COPD
Asthma
Allergic bronchopulmonary aspergillosis
Chronic bronchitis
Lymphocytic interstitial pneumonitis
Sjorgen's syndrome
Rhinosinusitis
Systemic lupus erythematous
Yellow nail syndrome
Ulcerative colitis
Coeliac disease
Crohn's disease
Graft versus host disease

**Table 3:** Defects in immune system as risk factors for the development of bronchiectasis.

Common variable immunodeficiency (CVID)	
IgG subclass deficiencies	
Alteration in memory B cells	
Defect in transporter associated with antigen presentation (TAP)	
HLA deficiency	
Hyper IgE syndrome	
Secondary immune defect (post-chemotherapy)	
Neutrophil oxidative burst deficiency	
Chronic granulomatous disease inability of PMN to produce superoxides	
Low mannose-binding lectin levels	
